# ResnetAge: A Resnet-Based DNA Methylation Age Prediction Method

**DOI:** 10.3390/bioengineering11010034

**Published:** 2023-12-28

**Authors:** Lijuan Shi, Boquan Hai, Zhejun Kuang, Han Wang, Jian Zhao

**Affiliations:** 1 Key Laboratory of Intelligent Rehabilitation and Barrier-Free for the Disabled (Changchun University), Ministry of Education, Changchun University, Changchun 130012, China; shilj@ccu.edu.cn (L.S.); 210401106@mails.ccu.edu.cn (B.H.); 2 Jilin Provincial Key Laboratory of Human Health Status Identification & Function Enhancement, Changchun 130022, China; 3 The Institution of Computational Biology of Northeast Normal University, Changchun 130000, China; wangh101@nenu.edu.cn

**Keywords:** DNA methylation, CpG sites, age prediction, deep learning

## Abstract

Aging is a significant contributing factor to degenerative diseases such as cancer. The extent of DNA methylation in human cells indicates the aging process and screening for age-related methylation sites can be used to construct epigenetic clocks. Thereby, it can be a new aging-detecting marker for clinical diagnosis and treatments. Predicting the biological age of human individuals is conducive to the study of physical aging problems. Although many researchers have developed epigenetic clock prediction methods based on traditional machine learning and even deep learning, higher prediction accuracy is still required to match the clinical applications. Here, we proposed an epigenetic clock prediction method based on a Resnet neuro networks model named ResnetAge. The model accepts 22,278 CpG sites as a sample input, supporting both the Illumina 27K and 450K identification frameworks. It was trained using 32 public datasets containing multiple tissues such as whole blood, saliva, and mouth. The Mean Absolute Error (MAE) of the training set is 1.29 years, and the Median Absolute Deviation (MAD) is 0.98 years. The Mean Absolute Error (MAE) of the validation set is 3.24 years, and the Median Absolute Deviation (MAD) is 2.3 years. Our method has higher accuracy in age prediction in comparison with other methylation-based age prediction methods.

## 1. Introduction

One of the leading challenges in aging research is measuring age accurately [1], which is also an essential forensic problem in determining the actual age (age calculated according to date of birth) of a sample [2]. DNA methylation, particularly 5-methylcytosine (5mC), has emerged as one of the most effective biomarkers for predicting biological age (the age determined based on the physiological status and functions of individual cells, tissues, and organ systems) [3,4,5]. DNA methylation is an important epigenetic modification that can have widespread effects on gene expression and cellular function. Changes in DNA methylation are influenced by genetic, environmental, and age factors. These changes can have an impact on disease and health, mainly due to gene expression regulation, disease risk, and environmental adaptability. Understanding the impact of these factors on DNA methylation will help us deeply understand the mechanisms of disease and provide new strategies and targets for disease prevention and treatment. Current age estimations are often performed based on DNA methylation level modeling, i.e., ‘epigenetic age (Epigeneticage)’ or ‘DNA methylation age (DNA methylationage)’ [6]. Epigeneticage acceleration (EAA) is the difference between DNA methylationage and actual age, and the positive or negative value of EAA represents the magnitude of DNA methylationage relative to actual age, indicating the exact aging degree of the individual. Due to the different living environments, tissue aging rate, and health status of individuals, there may be significant differences in the aging status group of individuals with the same actual age. So, exact age cannot comprehensively measure the aging status of the body.

In contrast, DNA methylationage cannot only measure whether an individual’s body function is in line with their actual age but also predict biological age and reveal the functional status of various body systems [7,8,9]. The Danish Twin Study found that the ability of DNA methylationaging prediction was significantly better than the perceived age (the estimated age based on rough visual observation) [10]. DNA methylationage has unique advantages over actual age, biological age, and perceived age in measuring the human aging process. It can provide a scientific reference for predicting and assessing aging status.

The rapid development of biological high-throughput technologies enabled the investigation of the aging process from different ‘omics’ perspectives [11,12,13]. Methylation represents an epigenetic modification type under the regulation of genetic elements and environmental factors. Methylome has been widely used to detect the chronological age from forensic samples [14,15,16,17,18] based on the observations of the age-related hyper- or hypo-methylated CpG sites (AR-CpGs) [19,20].

A classical epigenetic clock was first developed by Horvath et al. in 2013 [6]. The model’s training dataset included 8000 samples covering 51 healthy tissues and cell types. The correlation (R2) between DNA methylationage measured by the Horvath epigenetic clock and actual age was as high as 0.96, and the MAD between DNA methylationage and actual age was only 3.6 years. Although the Horvath epigenetic clock has some limitations in predicting the age of DNA methylation in some specific tissues (such as fibroblasts), it has gained popularity in the field due to its wide application and high accuracy of prediction results. Hannum’s epigenetic clock was developed in the same year by Hannum et al. [21]. This clock utilizes 71 age-related CPG sites for age prediction with a root mean square error (RMSE) of 3.9 years between its DNA methylationage and actual age. Compared with the Horvath epigenetic clock, the Hannum epigenetic clock is heterogeneous in inferring individual age results in non-adult and non-whole blood samples. Still, the DNA methylationage prediction accuracy is higher in adult blood samples. In 2019, Zhang Qian et al. constructed the ZhangAge age predictor [22]. Illumina 450K chips and Illumina 850K chips were collected as datasets, mainly from blood and saliva. The experimental results showed that the RMSE of this age predictor was 2.04 years. The above epigenetic clocks are all methylationage predictions using linear regression, and these models select a set of CpG sites that show a monotonic increase in age in a given training dataset, presenting a simple linear relationship [23,24,25]. Actually, there is no single linear relationship between DNA methylation and age, which may lead to the problem of high prediction error.

In recent years, deep learning models have been successfully applied to transcriptomics and clinical blood biomarker data for age prediction [26,27]. Galkin et al. showed their deep neural network model DeepMAge had slightly better predictive performance than the Horvath clock in blood samples in their 2020 study [28]. In the same year, Levine et al. developed a deep learning model, the MethylNet clock, and research has demonstrated that it has significant advantages over machine learning models [29]. A deep neural network model, AltumAge, was developed by Lapierre et al. in reference to DeepMAge in 2022 [1]. The mean absolute error (MAE) of the model’s prediction results was 2.153 years. Compared with linear regression methods, AltumAge has a great improvement in prediction accuracy, but the generalization ability of this model on independent datasets is found to be not good enough. So, there is room for further optimization of this model.

In this study, we proposed a method to construct ResnetAge based on the Resnet model, using 22,278 CpG sites common to both Illumina 27K and Illumina 450K arrays for DNA methylation age prediction. Compared with the model using the linear regression method for age prediction, our method has higher prediction accuracy, higher generalization ability, and interpretability after outputting via convolution of the Resnet model.

## 2. Materials and Methods

### 2.1. Data Source and CpG Site Selection

The datasets used in this experiment were obtained from the GEO (Gene Expression Omnibus) database, i.e., the high-throughput gene expression (www.ncbi.nlm.nih.gov/geo/) (The access date was 1 December 2022). The database includes high-throughput gene expression data submitted by research institutions around the world, mainly expression chip data, which contains methylation data. The 40 DNA methylation datasets used in this experiment are all derived from the Illumina platform Infinium HumanMethylation27 (27K) and Illumina Infinium HumanMethylation450 (450K). A total of 11,933 samples are included, with an age range of 0.17 to 114 years. Among them, 32 datasets are used for model training, and the remaining 8 are used as independent testsets to compare with three other methylation-based age prediction methods.

Zhang Qian et al. constructed the ZhangAge Predictor based on DNA methylation samples used for training selected from 13,661 samples (13,402 from blood and 259 from saliva) [22]. They showed that the epigenetic clock can be improved by increasing the training sample size and that its association with mortality attenuates with increased prediction of chronological age. Numerous studies have shown that CpG sites highly correlated with age tend to play a greater role in predicting age. That is, this site will have a higher weight in the model. For example, the Horvath epigenetic clock identifies 353 highly age-related CpG sites, while the Hannum epigenetic clock selects 71 CpG sites. The number of CpG sites in the standard 27K and 450K array data are 27,578 and 485,577, respectively, but there are missing CpG sites in many datasets, and the CpG sites of the 450K array do not wholly contain all the CpG sites of the 27K array. Therefore, a total of 22,278 CpG sites overlap in the 27K and 450K arrays were selected for model training in this experiment.

### 2.2. Data Processing

**Figure 1:**
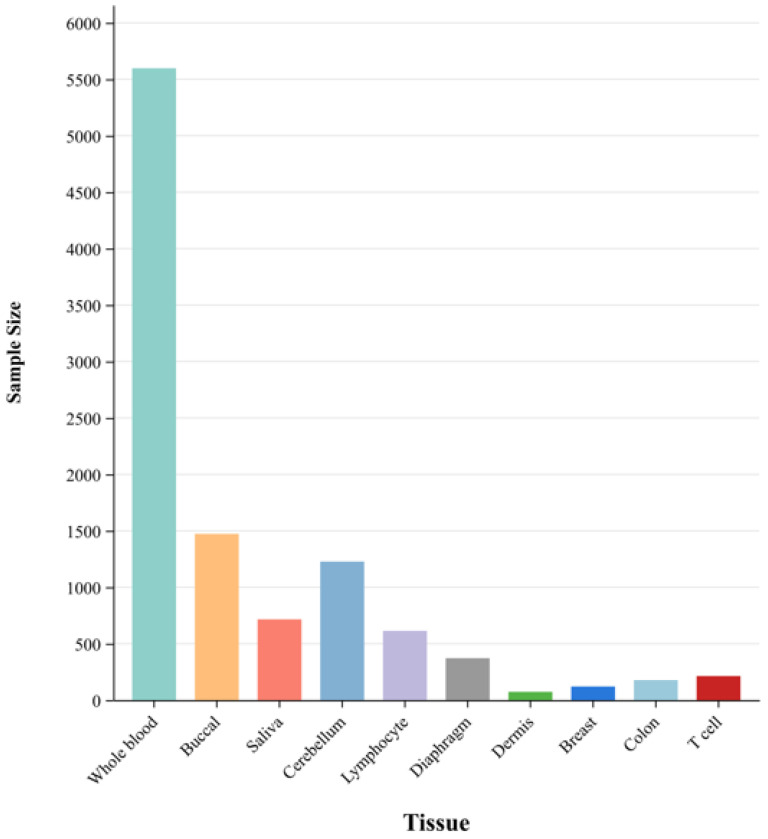
Organizational chart of the data sample. The figure divides the dataset into ten parts according to the organization, with whole blood data and buccal data accounting for a larger share.

In this experiment, methylation data were obtained by downloading GSE (Gene Expression Omnibus Series) data from the GEO database. For the downloaded data, normalization is performed first, and each individual GSE data is divided into two parts: the header file and the expression matrix. The header file part accesses various information about samples, including race, country, age, and collection organization. The expression matrix contains the CpG site values of all samples, that is, DNA methylation information. In addition, due to the problem of missing data in some datasets in the GEO database, it is necessary to remove samples with missing ages in the downloaded datasets to ensure that all samples contain true age values. Then, data with missing values in the expression matrix are imputed using the methyLImp method [30]. Finally, the DNA methylation data corresponding to the 22,278 CpG sites required for the experiment are extracted for all datasets. Figure 1 shows an overview of the 32 datasets by the organization, and all datasets information is provided in the Supplementary Materials.

### 2.3. Model Design

**Figure 2:**
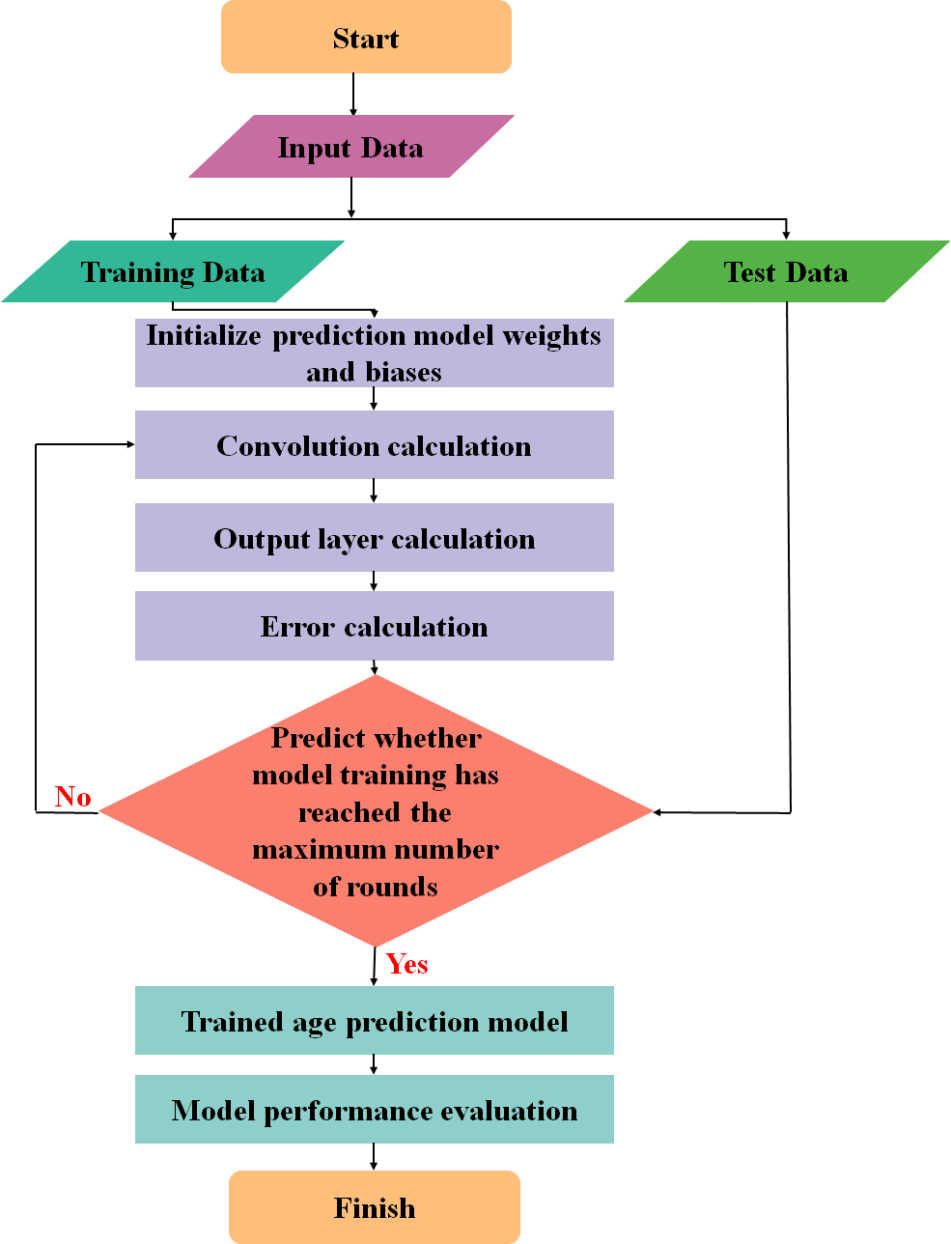
Overall flow chart of the model experiment.

In order to build and test the accuracy of ResnetAge, the overall flow chart of the model experiment was designed, as shown in Figure 2.

This experiment builds ResnetAge based on the Resnet model. The Resnet model can better learn residual information by introducing residual blocks, thereby improving the performance and training effect of the model [31]. Each residual block consists of a series of convolution layers, which include two main convolution layer sequences: identity mapping and feature mapping. As for the identity mapping, the input directly passes the information to the output by skipping the computation of some convolution layers to maintain the integrity of the information. However, for the feature mapping, the input performs a series of convolution operations to extract higher-level feature information. By combining the identity mapping and the feature mapping, the residual block can better learn the residual between the input and the output, allowing information to spread and update better. The input of the residual block, the output, and the feature mapping in the middle can be denoted as *x*, $H\left(x\right)$, and $F\left(x\right)$, respectively. Then, the output of each residual block can be expressed as $H\left(x\right)=x+F\left(x\right)$.

**Figure 3:**
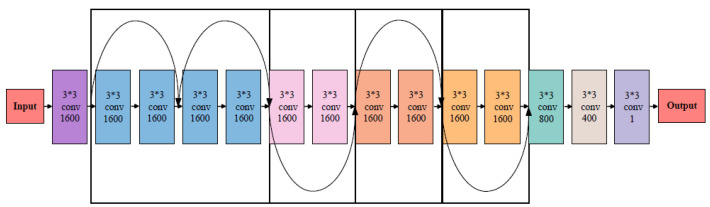
Model architecture. The model has 14 convolution layers and 5 residual blocks, each of which consists of two convolution layers. In the model, the beta value of DNA methylation is used as input, and feature extraction is performed through 14 convolution layers. Finally, the 4D data are transformed into one-dimensional data to output the predicted age values.

Figure 3 is a network architecture diagram of ResnetAge. The architecture is composed of 5 residual blocks, each containing two convolutional layers, along with a beginning convolutional layer and three tail convolutional layers, for a total of 14 convolutional layers. In the model, the beta value of DNA methylation of 22,278 CpG sites is used as input, and feature extraction is performed through 14 convolution layers. Finally, the number of model output channels is reduced to 1 to output the age value for prediction.

### 2.4. Model Training Process

The model designed in this experiment consists of 14 convolution layers. The initial data are one-dimensional data with a size of 1 × 22,278, and the size then becomes four-dimensional data with a size of 80 × 22,278 × 1 × 1 after being processed through the batchsize (set as 80 in the model) designed by the model and dimensional raising, which are correspondingly used as inputs for the model. The first convolution layer uses a 3 × 3 convolution kernel with a step size of 1, padding of 1, and an output channel number of 1600. Batch normalization is then performed, followed by activation of the function using ELU (Exponential Linear Unit). A stack of 5 residual blocks is performed afterward, and each residual block consists of two convolution layers and a skip connection. Each convolution layer uses a 3 × 3 convolution kernel with a step size of 1, padding of 1, and the number of output channels is 1600. Skip connections directly add the input to the output of the convolution layer. The output of each convolution layer goes through Batch normalization and ELU activation functions.

After passing the five residual blocks, the output channel number is decreased from 1600 to 800 through a convolution layer, and Dropout is added to discard neurons randomly after passing through the ELU activation function to prevent model overfitting. Then, after two layers of convolution, the output data size becomes 80 × 1 × 1 × 1, which is used as the age prediction value of the 80 samples in this batch. During the model training iterations, an ELU activation function is introduced in each convolution layer, given that DNA methylation data are not simply linear with age. At the same time, in order to prevent the data distribution from changing after the input data undergoes convolution processing, Batch normalization is added to each convolution layer to normalize the data.

## 3. Results Analysis

### 3.1. Evaluation Indicators

In order to evaluate the accuracy of ResnetAge, two evaluation indicators are used in this experiment: Mean Absolute Error (MAE) and Median Absolute Deviation (MAD), where MAE is shown in Formula (1):

(1) $$MAE=\frac{1}{m}\sum_{i=1}^{m}\left(predAge_{i}-trueAge_{i}\right),$$

The MAD is shown in Formula (2):

(2) $$MAD=median\left(\left|predAge-trueAge\right|\right)$$

In the formula, trueAge represents the actual age of the test data, and predAge represents the predicted age of the test data.

The MAE of the model on the training set is 1.29 years, and the MAD is 0.98 years; the MAE on the validation set is 3.24 years, and the MAD is 2.3 years.

### 3.2. Model Training Results

**Figure 4:**
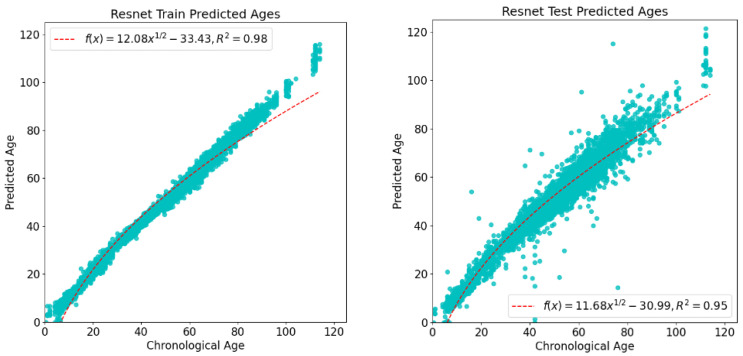
Model visualization metric results. The horizontal coordinates in the figure represent the actual age of the sample, and the vertical coordinates represent the model-predicted age. The left graph shows the prediction results of the model in the training data, and the right graph shows the prediction results of the model in the test data.

In this experiment, during the training process, the processed training dataset was randomly divided into training data and test data in a ratio of 7:3. The training data are used as a training set to train the model, and the test data are used as a validation set to verify the prediction accuracy of the model. Figure 4 illustrates the visualization of the model training results. The left graph shows the age prediction results in the training data, where the vertical axis represents the predicted age of the model, and the horizontal axis represents the actual age of the sample. The right graph shows the age prediction results in the test data. Similarly, the vertical axis represents the predicted age of the model, and the horizontal axis represents the actual age of the sample. As can be seen in most of the datasets, this experimental model performed well, with actual age and predicted age showing a high age correlation in the figure. By analyzing the model training results, it can be concluded that DNA methylationage is predicted in this experiment with high accuracy, and the prediction effect in each dataset is relatively stable.

### 3.3. Comparative Results of Different Methods

To verify the accuracy of ResnetAge for age prediction, the data array type used by the Horvath epigenetic clock is employed in the experiment. Additionally, the frequently used datasets in other epigenetic clocks are referred to, such as the Hannum epigenetic clock, etc. A total of 8 independent datasets (including three 27K and five 450K) were collected for testing. The predictions are compared with those of the Horvath epigenetic clock and other clocks. The comparison results are shown in Table 1.

As can be seen from the comparison, the prediction results of most independent datasets on ResnetAge are more accurate than those of the Horvath epigenetic clock, Hannum epigenetic clock, and ZhangAge predictors. Since Hannum and Zhang et al. did not use Illumina 27K array data in their model training, the test results of their method on the 27K dataset are not recorded in the comparison results. At the same time, the bold font indicates that the performance of ResnetAge under this indicator is better than the method corresponding to the row name.

From the comparison results with the Horvath epigenetic clock, it can be seen that most of the test results of ResnetAge in the three27K array data and five 450K array data are mostly superior to those of the Horvath epigenetic clock, with only three datasets not performing precisely enough. The Illumina 27K chip has a total of 27,578 CpG sites. ResnetAge, in this experiment, selected 22,278 of them. Compared with the 353 CpG sites used by the Horvath epigenetic clock, the 27K data accounted for a larger proportion, so the test results were also better than those of the Horvath epigenetic clock. Although compared with the 485,577 Illumina 450K chips, the 22,278 CpG sites are not large, and the model has insufficient feature extraction ability for the data during training. Therefore, the performance of ResnetAge and Horvath epigenetic clocks on 5 datasets of 450K array datasets is not sufficiently precise.

Independent datasets GSE53740 and GSE30758 perform slightly worse. The analysis found that the tissues in the dataset GSE53740 are derived from whole blood, and some of the samples suffer from diseases such as Alzheimer’s, etc. It is well understood from the literature that disease causes aberrant DNA methylation levels and thus impacts age prediction. The datasets selected by ResnetAge during model training are all healthy samples, which leads to the insufficient feature extraction ability of this model for datasets containing diseases, so GSE53740 had a large error in prediction results. The tissues in dataset GSE30758 are mainly from normal cells of the uterine cervix, and ResnetAge does not select a dataset from this tissue during training, while the Horvath clock is a pan-tissue epigenetic clock (training samples from 51 tissues and cell types), which was well tolerated for tissue specificity, so the Horvath epigenetic clock has better results on GSE30758.

From the comparison results of the Hannum epigenetic clock, it is found that the performance of ResnetAge on the 450K array is mostly better than that of the Hannum clock. The Hannum epigenetic clock uses whole blood samples from 656 human individuals aged 19 to 101 years during the training process, so the prediction result of the Hannum clock is highly erroneous on partial non-whole blood datasets. Similarly, the prediction results of the same dataset, GSE53740, are slightly worse than those of the Hannum epigenetic clock, which is also caused by the fact that this dataset contains disease samples.

Compared with the ZhangAge epigenetic clock, it can be seen that the prediction results of ResnetAge are mostly better than those of ZhangAge. The sample tissues in the training set used by the ZhangAge epigenetic clock are mainly from blood and saliva, so there is a clear advantage to the test results of blood and saliva samples. The tissue type in the dataset GSE111223 is saliva, and the tissue type in the dataset GSE53740 is whole blood, so it is reasonable that the prediction results of these two datasets are slightly worse than those of ZhangAge.

### 3.4. Prediction Performance in Different Tissues

**Figure 5:**
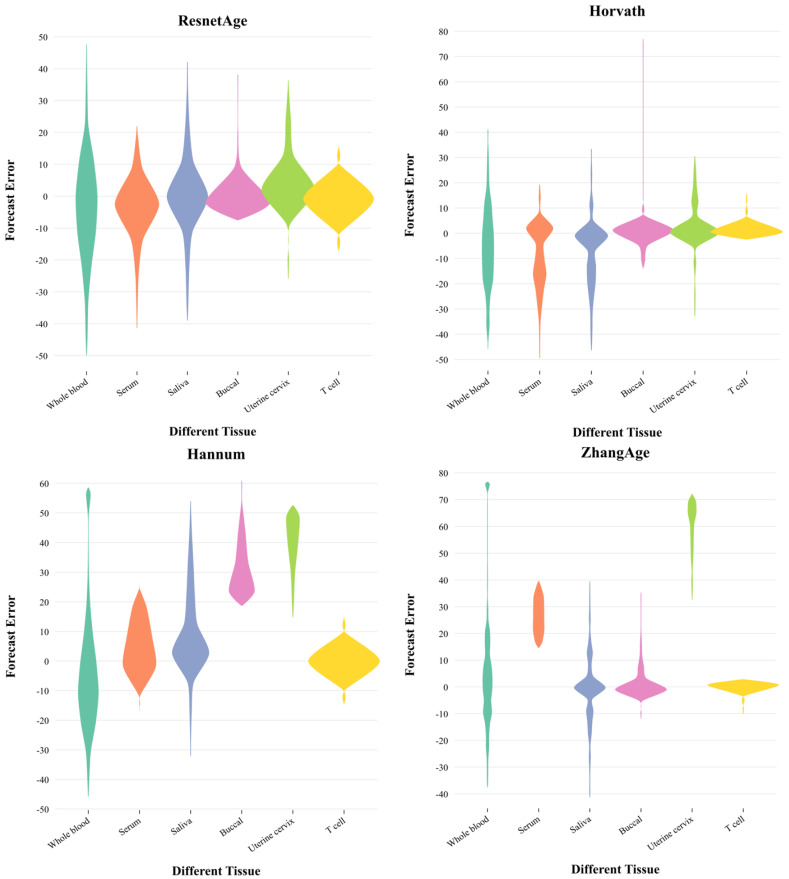
Prediction errors of different methods in six organizations. The four violin plots represent the methods of ResnetAge, Horvath, Hannum, and ZhangAge, respectively. The six colors in each figure represent the age prediction errors of six types of tissues: whole blood, serum, saliva, buccal, uterine cervix, and T cell. The age prediction error is calculated by subtracting the actual age from the predicted age. The width of the violin plot represents the data density of the prediction error. The wider part indicates that the data distribution is denser, and the narrower part indicates that the data distribution is sparse.

To further explore the prediction accuracy of ResnetAge, we also performed age predictions on different tissues in an independent dataset, and Figure 5 represents the prediction error of different methods in each tissue. The abscissa in the figure represents six different tissues: whole blood, serum, saliva, buccal, uterine cervix, and T cell, while the ordinate represents the prediction error, which is calculated by subtracting the actual age from the predicted age. Because the Horvath epigenetic clock, Hannum epigenetic clock, and ZhangAge predictor all use linear regression for methylationage prediction, this leads to some limitations in their methods, and the prediction error is high in these tissues. In contrast, our ResnetAge is built based on a deep learning model, which can better acquire feature information. A comprehensive analysis of the distribution contours and distribution areas of each method in the violin plot can be used to conclude that the prediction results of ResnetAge are relatively stable in various organizations, the prediction errors are densely distributed around 0, and the error span is relatively small, which further shows that ResnetAge can improve age prediction accuracy.

## 4. Conclusions

With the continuous development of the field of aging materiology, new methods for quantitative measurement of aging continue to emerge. Among them, the epigenetic clock is a tool that helps researchers better understand and measure the aging process. Due to the good performance of deep learning models in dealing with unstructured data, they have shown great promise in many biological tasks. Deep learning models not only represent an improvement in the performance of current epigenetic clocks but also provide new and relevant biological insights into the aging process. In the medical field, our method can better explore the biological mechanisms of aging and degenerative diseases by predicting biological age and promoting the healthy development of aging.

A new age predictor based on the Resnet model, ResnetAge, is presented in this paper. Compared with the previous penalty regression model, ResnetAge has a stronger feature extraction ability, which can improve the accuracy of age prediction. The results of the independent test set show that ResnetAge has high prediction accuracy on most datasets, except for certain limitations on datasets containing disease information. ResnetAge provides a new feasible idea for the field of epigenetic clock and aging and also provides a new deep learning-based solution for this field.

ResnetAge has the potential for further improvement. Because the DNA methylation dataset selected in this model during training consisted of all healthy samples and the tissue types are not fully diversified, it can also be seen from the results of comparative experiments that some datasets have large errors. Referring to domestic and foreign literature, it is found that the effect of different diseases on DNA methylation varies. So, in future work, we will also consider the disease information of samples in the dataset. To enable further exploration of disease in relation to DNA methylation, subsequent experiments will also focus more on datasets containing disease samples. In order to enable a more comprehensive and accurate prediction of pan-tissue DNA methylationage, more samples of different types will also be added in the subsequent data processing. Accurate epigenetic clocks can assist clinicians in judging whether age-related diseases occur and will better help clinicians make a diagnosis, which is of great significance for precision medicine.

## Figures and Tables

**Table 1:** Comparison Between ResnetAge and Other Clock Results (Bold font indicates that ResnetAge performs better than the row name corresponding method under this indicator). (∖ indicates that no comparison is made here because Hannum [21] and Zhang et al. [22] did not use 27K arrays of data for training in their models).

Dataset	Platform	MAE ResnetAge	MAD ResnetAge	MAE Horvath	MAD Horvath	MAE Hannum	MAD Hannum	MAE ZhangAge	MAD ZhangAge
GSE57484	27K	6.93	6.62	**8.63**	**8.88**	\	\	\	\
GSE58119	27K	8.39	6.77	**13.99**	**12.84**	\	\	\	\
GSE137495	450K	0.26	0.25	**0.59**	**0.56**	**26.22**	**26.2**	**1.31**	**0.94**
GSE80261	450K	4.39	3.34	3.84	3.14	**36.24**	**35.93**	**4.74**	**4.48**
GSE111223	450K	10.48	8.48	**10.54**	**9.73**	**12.08**	**11.07**	0.9	0.78
GSE71245	450K	8.17	5.44	7.17	**5.67**	**10.27**	**9.29**	5.44	**6.14**
GSE53740	450K	12.02	9.9	8.22	7.28	8.82	8.43	0.48	0.49
GSE30758	27K	11.89	10.9	7.2	7.11	\	\	\	\

## Data Availability

The dataset used in this study comes from the Gene Expression Omnibus (GEO) database, a publicly available comprehensive repository of gene expression, which can be downloaded from: https://www.ncbi.nlm.nih.gov/geo/ (The access date was 1 December 2022).

## Supplementary Materials

The following supporting information can be downloaded at: https://www.mdpi.com/article/10.3390/bioengineering11010034/s1, The Excel file named “Datasets” provides supplementary material, which includes all datasets used for model training. The sources and download addresses of these datasets are stated in the Data Availability Statement. The code for the ResnetAge model is located at: https://github.com/52hai/ResnetAge (The access date was 1 December 2022).
